# Acute anodal transcranial direct current stimulation improves the performance of professional rowers

**DOI:** 10.3389/fspor.2024.1310856

**Published:** 2024-04-18

**Authors:** Luciano Ramos, Tatiana Aparecida Magacho Ramos, Rodrigo Freire De Almeida, Jader Vinicius da Silva-Rocha, Carla Zimerer, Fernando Zanela Arêas

**Affiliations:** ^1^ Physiotherapy Course at the FAVI—Victorian Higher Education Association, Vitória, Brazil; ^2^ Neuromodulation Institute, Vitória, Brazil; ^3^ Group of Study and Research in Neurorehabilitation and Neuromodulation, Federal University of Espirito Santo, Vitória, Brazil; ^4^ Postgraduate Program Physiological Sciences, Center of Health Science, Federal University of Espirito Santo, Vitória, Brazil; ^5^ Postgraduate Program in Electrical Engineering, Federal University of Espírito Santo, Vitória, Brazil; ^6^ Telecommunications Laboratory, Federal University of Espírito Santo, Vitória, Brazil; ^7^ Physiotherapy Course at the Federal University of Espirito Santo, Vitória, Brazil

**Keywords:** ergogenic aid, neuromodulation, tDCS, insula, sports, performance, fatigue, rowing

## Abstract

**Introduction:**

The aim of the present study was to evaluate the influence of acute transcranial direct current stimulation (tDCS) on physical and subjective responses in professional rowing during the 2,000-m time trial test.

**Methods:**

Seven rowers (age 20.86 ± 4.49 years; weight 71.66 ± 7.97 kg) participated in this randomized triple-blind trial with a crossover experimental design. The protocol consists of 2 days with different conditions (anodal and sham). The tDCS anodic stimulation conducted was 2 mA for 20 min in the left temporal cortex (2.5 cm from the F7 zone and 2.5 cm from the T3 zone), targeting the left insular cortex. In the sham moment, the participants experienced 30 s of stimulation. Afterward, they performed a standardized progressive warm-up for 15 min, following the Brazilian Rowing Confederation's assessment protocols, and rested for 3 min before the test started. All procedures were made on an indoor rowing machine, which allowed the capture of performance variables such as time performed, power in watts (W), pace (m/min), and stroke rate (strokes/min). The ratings of perceived exertion [Borg scale (CR-20)] were recorded in each 2-min during the test.

**Results:**

The results presented differences in power [Z: −2.371; *p* = 0.018; effect size (ES) = −0.896 (large)] and pace [Z: −2.371; *p* = 0.018; ES = −0.896 (large)] and time performance [Z: −1.612; *p* = 0.107; ES = −0.609 (large)] throughout the protocol for the anodal moment.

**Discussion:**

However, no differences for the other variables were found. According to the results, the current tDCS with the present protocol improved the physical performance at the 2,000-m time trial Test providing ergogenic aid.

## Introduction

Rowing is a sport where an individual or group propels a boat on water using oars. In a 2,000-m course rowing competition, the rank is decided based on the finishing order. It lasts 6–8 min, with several muscle groups being involved (legs at approximately 65%, back at approximately 25%, and arms at approximately 10%) (1–4). For rowers to improve their records and win medals, highly developed aerobic and anaerobic systems are required (4, 5). Hence, rowers train to improve not only aerobic and anaerobic capacities but lactate tolerance, strength, and power as well as performance (4, 6, 7).

Recent studies have observed that central elements of the brain, including the cortical regions, play an essential regulatory role in endurance performance (8). They have linked the brain regions’ excitability with cognitive–motor control, leading to the neural drive output augment and spinal motor neuron excitability. This effectively increases endurance during exercise and alleviates fatigue (9–13). Fatigue is a complex, multifactorial physical and perceptual experience of intense and sustained activity endurance that involves muscles and the nervous system (10–12).

This symptom can reduce muscular performance, decrease force, and impair response time and decision-making skills (10). It may arise not only because of peripheral changes at the level of the muscle but also because the central nervous system (CNS) fails to drive the motoneurons effectively (10). Central fatigue, i.e., fatigue in the CNS, presents during exercise, a progressive change in the corticospinal-motoneuronal pathway, which leads the neural drive from higher brain areas to the exercising limb muscles (14). Those properties are inhibited/reduced in their action, leading to the voluntary drive for muscle activation (14). Thus, the primary brain area related to the sensation of sports fatigue is the insular cortex (IC) region (11, 12, 15), which should be visualized through a complex systems understanding (16).

Therefore, strategies designed to facilitate the excitability of cortical regions within the brain seem to enhance endurance capacity, which may help to improve sports performance by generating an ergogenic effect (14, 17, 18). This hypothesis is already being tested in some sports, and several studies have shown improvement in a neuromodulatory technique called transcranial direct current stimulation (tDCS) (19, 20). Among these ergogenic strategies, tDCS is a technique that can non-invasively and safely modulate cortical activities by sending direct micro-level currents to the targeted regions via scalp electrodes (17, 21). Consequently, previous studies have revealed that fatigue perception is reduced after tDCS anodal intervention, which can be credited to an improvement in sports performance (14, 22).

In recent years, more attention has been paid to the ergogenic effects of tDCS on endurance performance (17, 21). For example, Vieira et al. (23) showed that using tDCS to modulate the prefrontal cortex's cortical excitability can significantly improve sports endurance performance in back squats and swimming. In another study, Wang et al. (24) reported that bilateral modulating the cortical excitability of the primary motor cortex (M1) using tDCS can significantly improve muscle endurance performance in elbow flexion tasks.

Recently, studies involving tDCS intervention in rowers showed promising results. Liu et al. (22) conducted the first study to explore the mechanism of tDCS on the performance of rowers. The authors found an improvement in executive function and performance at the indoor rowing ergometer test after performing tDCS sessions in the left motor cortex (LM1) five times a week for 2 weeks in two groups of professional rowers. Liang et al. (25) obtained results from a pilot study that provided valuable preliminary insight into the design of protocols for future studies examining the effects of the Halo Sport System of tDCS on the endurance of elite rowers. The authors found an improvement in performance results with bilateral anodal stimulation placed over the CZ area (CZ-M1) and bilateral cathodal stimulators placed over C5 and C6. The group found improvements in time and power performance during the 5,000 m test over 3 days. Ma et al. (26) conducted the first study to explore the central mechanisms of tDCS in improving the performance of male rowers from the perspective of regional brain activity and whole-brain functional networks. For this, the authors stimulated the LM1 with tDCS for 20 min, five times per week for 2 weeks during regular training sessions. They analyzed their resting state using functional magnetic resonance imaging (MRI), finding that simultaneous tDCS-induced excitation of the M1 could improve the overall performance of male rowers.

However, the literature indicates that results about the effects of tDCS on performance are controversial. For example, Alix-Fages et al. (27) evaluated three 15-min sessions of tDCS (anodal, cathodal, and sham) in the dorsolateral prefrontal cortex in 25 men and concluded that stimulation did not improve sprint performance or ratings of perceived exertion. On the other hand, Shyamali Kaushalya et al. (20) demonstrated that anodal tDCS enhances running and cycling time to exhaustion (TTE) performance. These results may have occurred due to differences in the study methods, such as voltage, stimulation time, target brain area stimulated, or studied population.

Nevertheless, to the best of our knowledge, the investigations of the acute effects of tDCS in rowers have yet to be well characterized. Thus, the aim of the present study was to evaluate the influence of 20 min of anodic tDCS on physical and subjective responses in professional rowers during the 2,000-m time trial test. Based on the available evidence regarding the role of tDCS on performance in rowers, the hypothesis of this study is that tDCS will increase time trial test performance and improve the perceived exertion scale compared to sham tDCS.

## Methods

### Participants

The researchers did a preliminary demographic analysis and collected information about professional rowing clubs in their local places in Brazil. It was found only two professional rowers' clubs in the Espírito Santo State. The first contact was made with the coaches. Later, all male rowers from both clubs were invited to participate in this study. Of the 18 professional rowers, seven decided to participate in this study (Table 1). The participants signed an informed consent form after a verbal explanation of the procedures carried out. All rowers were highly trained/national level (Tier 3), according to McKay et al. (28), and training was for approximately 12.38 h per week. All the participants were actively training (in a competitive period undergoing regular activity), with no injuries or illnesses, and were familiar with the procedures herein, counting with the rowing ergometer. The exclusion criteria included being injured or returning from injury, unsuitable rowing performance, or taking psychoactive medication. No rowers were excluded from the analysis. They were instructed to keep their food and daily routines. Individuals under 18 years of age participated with parental consent. The procedures were approved by the University Ethics Committee for Study in Humans (registration no. 02571718.0.0000.5068).

**Table 1:** Descriptive data of study participants.

	Age (years)	Body mass (kg^−1^)	Height (cm)	IMC (kg/m^2^)
Participants	20.86 ± 4.49	71.66 ± 7.97	178.26 ± 5.84	22.58 ± 2.41

Values are denoted as mean ± SD.

### Study design

A crossover, randomized, triple-blind trial was performed for the present study. The participants were fully informed of all processes, such as the following protocol. One week after, the familiarization session begins. Thus, they performed a rowing test exercise protocol described as follows: each participant was assigned a number and then randomly assigned to a group (anodal or sham) using a table of random numbers. The blinding process was carried out by a third participant who did not participate in the data collection. This participant programmed the tDCS machine in isolation from the tDCS applicator, the rowers, and the other researchers involved. Thus, all involved participants were blinded.

### Exercise protocol and study variables

Testing sessions were performed at the same time (8 a.m.) to avoid variations in the circadian cycle. The routine was close to the rower's routine training schedule. There was an interval of 72 h between sessions, as in the study by Liang et al. (25). Environmental conditions were kept the same (temperature: 21°C; absolute pressure of 1 atm: 14.7 psi).

The tests were carried out in the Laboratory of Physiology Applied to Sport of the Victorian College of Technology, campus of Vitória City, Brazil. After they arrived at the local test, they equipped and started the warm-up on the indoor rowing machine, which was used as an apparatus to perform the 2,000-m time trial test.

This is an accepted ergometer test, probably used in all elite rowing programs worldwide, where the rower aims to cover the virtual distance of 2,000 m as fast as possible (4).

The evaluation reproduces the Olympic Rowing competition (29) with good reliability, especially in small boats, where the result can be associated with the water performance outcome (7). Male and female elite rowers can finish the race in approximately ≤5:50 min and ≤6:50, respectively (4).

Before the test, a warm-up was done, adapted as requested by the Brazilian Rowing Confederation's assessment protocols (29). The participants rowed for 15 min, progressing from low intensity to high intensity. The rowers were asked to increase the stroke rate and power by doing 5–10 strokes per serie until reaching their maximum stroke for the test. After the warm-up, they had a passive recovery for 3 min before the test started.

**Figure 1:**
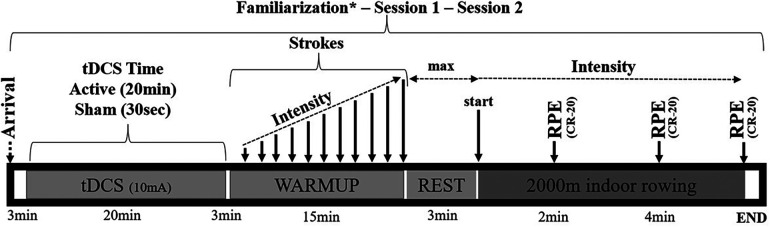
Experimental protocol. *Session to reproduce all procedures with no active or sham tDCS.

The 2,000—m time trial test allows the coaches to catch physiological and performance variables as well as other important information that can be acquired. This study assessed performance variables such as time completed (min), power (W), pace (m/min), and stroke rate (i.e., stroke/min in arbitrary units). In addition, the rating of perceived exertion [RPE (CR-20)] was taken every 2 min at the end of the test. RPE was measured using a scale of 6–20 (from “no exertion at all” to “maximal effort”), according to Borg (30). The researchers provided verbal instructions during all the protocols. During the test, they instructed the rowers to fulfill the objective, which was to row as fast and powerfully as possible, as a primary tool to improve their performance (3). They provided verbal encouragement, and at each point, as described in Figure 1, they collected the subjective variable until the end of the protocol. For hydration, water was consumed *ad libitum* before and after the protocol.

The performance variables and the RPE were assessed during and after the physical protocol. All participants completed the tests. No side effects or injuries were reported.

### 2,000-m time trial test, performance, and subjective variables

First, ergometers play an essential role in rowing training and are used to select crews (31). The power produced may be a determining factor in performance (31). However, the problems with on-water testing have led to the widespread use of the Concept 2 rowing ergometer (32) for home-based training, and performance assessments in sports and science (4, 32). Thus, in this study, the RowErg rowing ergometer with a PM5 monitor by Concept2 (Morrisville, VT, USA) provides accurate estimates of a rower's physiological ability to output power and some submaximal and brief maximal ergometer performance measures that can be used to monitor changes in this ability (32).

Nevertheless, the 2,000-m time trials were performed on the indoor rowing machine, considered the gold standard for performance testing (32–34). Thus, it is a valid, accepted ergometer test, probably used by all elite rowing programs worldwide, where the rower aims to cover the virtual distance of 2,000 m as fast as possible (4). A positive relationship exists between the 2,000-m rowing ergometer performance and the 2,000-m on-water performance, which correlates with their position in their final World Rowing Championships rankings (7).

Previous studies about rowers showed that time, power, pace, and stroke rate are essential variables to be understood, taken, and analyzed to aim for improvement in the rowers’ performance (4, 35). Those parameters are valuable to the coaches (35).

The subjective variable used was the RPE scale developed by Borg (30). The Borg CR-20 scale is a classic tool used in several areas, such as rehabilitation and endurance training. RPE scales are of great value in capturing participant information, such as physiological variables (30). Previous studies about rowers have demonstrated that RPE correlates with heart rate (HR) and blood lactate during tests (4, 36, 37).

### Transcranial direct current stimulation

Before the 2,000-m time trial test, the participants were submitted to the tDCS administered by a battery-powered stimulator (neuroConn, Ilmenau, Germany) through a pair of silicone electrodes (size: 6 cm × 8 cm, two 35 cm^2^) wrapped in a sponge soaked in saline liquid (9% NaCl).

The tDCS was carried out using the following procedure: the individuals were divided randomly into the anodal (20 min of anodal stimulation) or sham groups (30 s of anodal stimulation and the rest with no stimulation) (18, 38). The electrodes were positioned on the participants’ heads and cleaned with neutral soap before coupling. Then, two electrodes were wrapped in specific places on the participant's scalp in the respective regions of interest using the international mapping system EEG 10–20 (39).

The electrode for anodic stimulation was fixed in the left temporal cortex (LTC) (2.5 cm from the F7 zone and 2.5 cm to the T3 zone), targeting the left insular cortex (LIC) (40) for 20 min of anodic stimulation (41, 42). The LIC was hypothesized to be an area related to an athlete's fatigue sensation (12). Considering the connections between the temporal cortex and LIC, it has been shown, by computational modeling and experimental studies, that tDCS applied over the LTC probably modulates the activity of the LIC, resulting in increased parasympathetic modulation at rest and during exercise, although the LIC is a relatively deep brain structure (43). This study wanted to modulate this area to increase performance by decreasing the central sensation of fatigue and exercise-induced pain (43). Therefore, the electrode for cathodic stimulation was positioned in the right contralateral supraorbital area (Fp2 area), a reference in several studies (18).

Finally, the electrodes were connected to a constant electric current stimulator device with three power batteries connected in parallel (9 V), with an energy output of up to 10 mA as used in sports research (18) as the effectiveness of the acute anodic tDCS session (44–48).

## Statistical analysis

All data were expressed as descriptive statistics (mean ± standard deviation). The inferential statistical analysis was performed using the Shapiro–Wilk test to verify the data distribution and the homoscedasticity test (Bartlett's criterion). All the analyzed variables did not show a normal distribution or homoscedasticity. The Wilcoxon signed-rank test was used to verify the difference between the two moments, adopting a significance level of *p* ≤ 0.05. As effect size (ES), we used the *r*-value from the paper by Cohen (49) as orientation. The ES reference values are considered to be 0.2, 0.3, and 0.5 for small, moderate, and large ES, respectively. Large and moderate ES are relevant in the context of determining clinical differences and are applied according to Lakens (50). The software used was SPSS v.21 (IBM Corp., Armonk, NY, USA), and GraphPad Prism v.8.0 was used to generate graphs.

## Results

Consequently, according to the Table 2, the Wilcoxon signed-rank test detected differences between conditions for power [*Z*: −2.370; *p* = 0.017; ES = −0.896 (large)] and pace [*Z*: −2.371; *p* = 0.018; ES = −0.896 (large)], indicating an increase in performance. It is possible to detect a clinical difference in the time performance [*Z*: −1.612; *p* = 0.107; ES = −0.609 (large)] and an antagonistic effect in the stroke rate [*Z*: −1.225; *p* = 0.221; ES = −0.463 (moderate)] as well as in the subjective scale (RPE CR-20) in moment 1 [RPE-1; *Z*: −0.843; *p* = 0.399; ES = −0.319 (moderate)] throughout the protocol.

**Table 2:** Group performance results.

Groups	Time (s)	Power (W)	Pace (min/m)	Stroke rate (AU)	RPE-1	RPE-2	RPE-3
Sham	1,354.69 ± 144.43	307.14 ± 55.73	18,428.57 ± 3,344.32	31.28 ± 1.60	12.42 ± 1.90	15.00 ± 1.0	17.42 ± 1.39
Anodal	1,045.71 ± 83.83	318.85 ± 54.15	19,131.42 ± 3,249.50	30.57 ± 1.13	13.28 ± 2.05	15.14 ± 2.11	17.85 ± 1.86

AU, arbitrary unity; RPE-1, rating of perceived exertion for the first moment; RPE-2, for the second moment; RPE-3, for the third moment.

All RPEs were based on Borg Scales 0–20.

**Figure 2:**
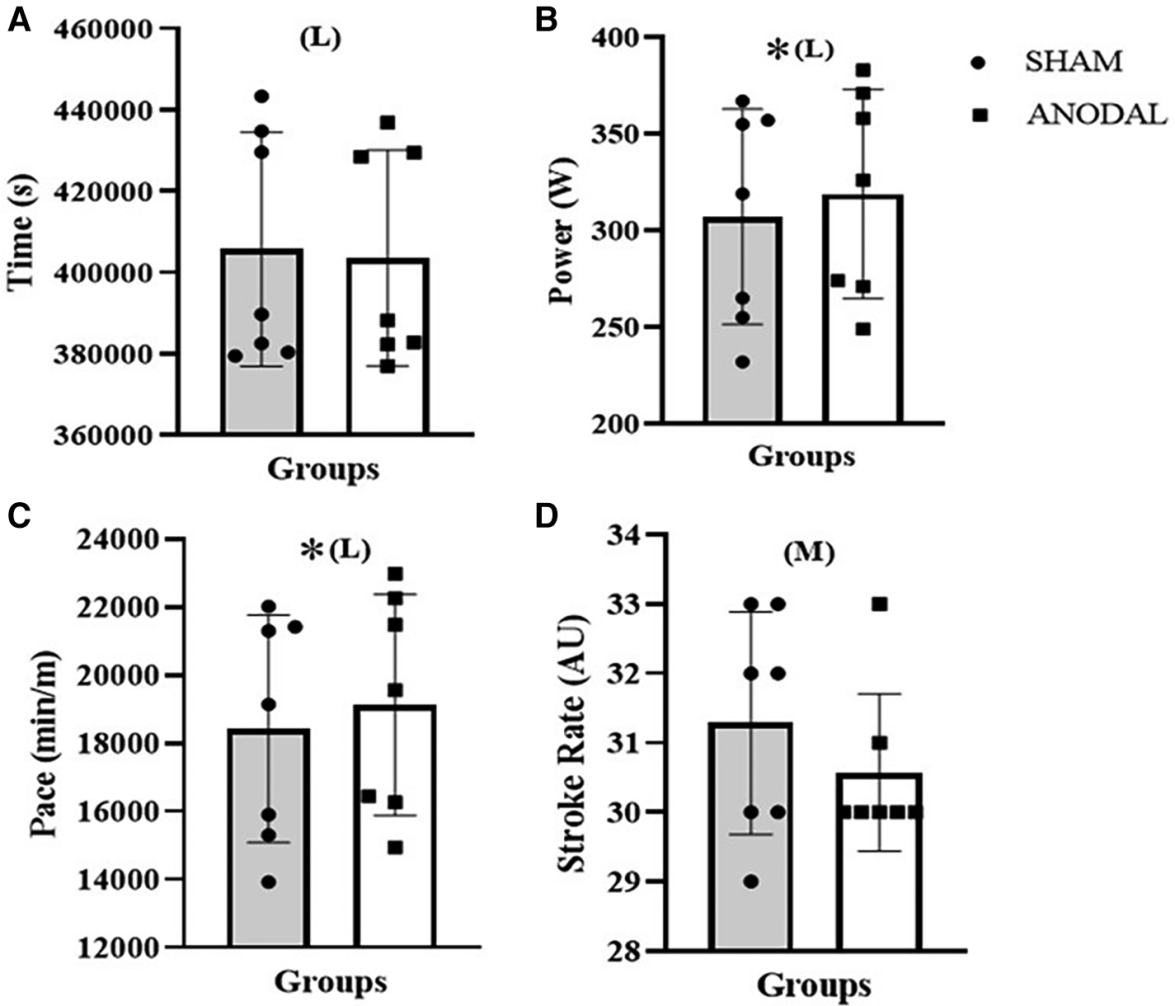
Time (**A**), power (**B**), pace (**C**), and stroke rate (**D**). *: *p* < 0.05. AU, arbitrary unity; L, large effect size; M, moderate effect size; S, small effect size.

**Figure 3:**
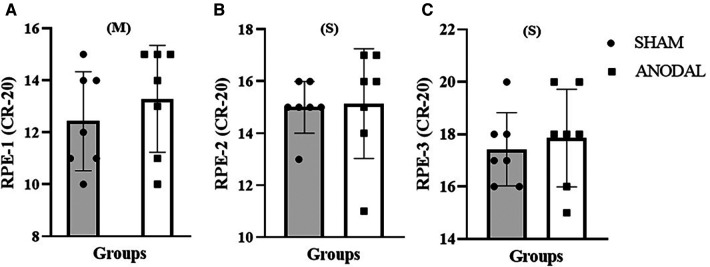
(**A**) Rating of perceived exertion (Borg CR-20) for the first moment (RPE-1); (**B**) rating of perceived exertion (Borg CR-20) for the second moment (RPE-2); (**C**) rating of perceived exertion (Borg CR-20) for the third moment (RPE-3). M, moderate effect size; S, small effect size.

However, in RPE moment 2 [RPE-2; *Z*: −0.276; *p* = 0.783; ES = −0.104 (small)] and moment 3 [RPE-3; *Z*: −0.756; *p* = 0.450; ES = −0.286 (small)], there were no differences, according to Figures 2 and 3.

## Discussion

The aim of the present study was to evaluate the influence of tDCS on physical and subjective responses in professional rowers during the 2,000-m time trial test. Thus, after 20 min of tDCS, rowers exhibited better performance considering variables such as time, power, and pace. This permit verifies the ergogenic effect of tDCS in rowing practice. Conversely, regarding the stroke rate and RPE (CR-20) in moment 1, it is possible to detect adverse effects between the conditions, i.e., indicating a placebo effect. To our knowledge, the present study was the first to apply tDCS in the LIC area, aiming to improve the physical and subjective performance of male professional rowers during the 2,000-m time trial test.

Despite all considerations, rowers require highly developed aerobic and anaerobic systems as well as significant strength and power (4–6). The 2,000-m time trial is a high-demand, hugely exhaustive test (4). To accomplish this task, the present study used highly trained participants (28).

Hence, highly trained female rowers showed a strong correlation between time and power over 2,000 m with an average speed of over 2,000 m (35). They confirmed that the force applied in each stroke influences performance in international rowing competitions. In addition, the technical behavior related to this kind of rowers’ level correlated with power is a crucial factor in the female rowers’ world championships. However, in the present study, the participants were compared with themselves. The tDCS protocol can explain the possible difference.

Further, the stroke rate of the elite rower, anywhere from 1,000 m in the 2,000-m race, seems crucial in real competitions (3). The authors mentioned that elite rowers from the New Zealand National Rowing Team (NZNT) during the 2,000-m race did 34 strokes/min. In this study, highly trained Brazilian national rowers did approximately 31 and 30 strokes/min, respectively, in the sham and anodal groups. There is a slight difference of 9.68% and 13.33%, respectively, compared to the NZNT. The race-to-race variability in finish times for elite rowers in world cups, championships, and Olympic competitions is approximately 1% (2). Thus, considering the massive variance in this study, it is possible to speculate on the environmental differences between laboratory tests and actual competitions.

One study estimated the most negligible significant performance enhancement ( approximately 0.3%) and the effects of the level of competition, finals level, venue, environment, and boat class (2). Those races show maximal efforts for highly motivated and well-conditioned rowers (32). In this sense, elite rowers can experience stressful situations that demand an increase adaptive response to the challenge of competition than in the training situation (51). Nonetheless, more studies are necessary to disclose these environmental differences under tDCS intervention, other neuromodulation tools, or ergogenic aid in rowers.

As mentioned, Liu et al. (22) selected 12 participants and randomly placed them in two groups: one group of low-stimulation (1 mA) and another of high-stimulation (2 mA). Both groups did 10 tDCS sessions of 20 min five times a week over two consecutive weeks while undergoing regular training. They explored the anodic electrodes at the LM1 and the cathode electrode on the contralateral shoulder. Using the progressive incremental test, the participants presented significant enhancements to executive function (related to better well-being, faster recovery, and performance gains). In addition, increased performance was demonstrated by the highest peak torque of the right knee joint at 60°/s on the isokinetic dynamometer. The authors claim that improvements in sports performance may be due to other reasons. However, as in this present study, there were no significant changes in perceived effort. Rowers improved their performance under the same or slightly lower RPE result, showing significant tolerance to the imposed load under the tDCS anodal moment, as found in Angius et al. (18) and Okano et al. (52).

The study by Okano et al. (52) targeted the stimulation of the temporal and insular cortex (TC-IC), which is associated with autonomic nervous system (ANS) control and the awareness of feelings in the body. According to the authors, ANS relates to RPE responses in the regulation of exercise performance. The authors performed 20 min of anodal tDCS over the left TC-IC (T3) to confirm the effect on the ANS. They evaluated the RPE and performance variables during a maximal dynamic cycling exercise. The peak power output improved by approximately 4%, the parasympathetic vagal withdrawal was delayed, and the HR was reduced at submaximal workloads. The RPE increased slowly during exercise, but cortical stimulation did not affect maximal RPE or HR values. They suggest that tDCS over the TC-IC modulates the ANS activity and the sensory perception of effort and exercise performance.

In contrast to previous authors, Ciccone et al. (53) investigated the effect of the same T3-Fp2 tDCS montage on 50 repetitions of a maximal effort knee extension task in the isokinetic dynamometer protocol (a repeated maximal effort work capacity) and heart rate variability (HRV). The findings did not show differences in HRV variables or the physical task, which was in contrast to the results from the previous authors (52). There is no difference in the Fatigue Index between the physical protocols. Thus, anodal tDCS may only enhance exercise perception and performance at low exercise intensities before complete parasympathetic withdrawal occurs (54). In this sense, Barwood et al. (54) did not detect differences under the same tDCS conditions during the maximal test to determine the aerobic capacity undertaken during a 20-km cycling time trial on performance and RPE variables in a hot environment.

Nonetheless, as mentioned before, Liang et al. (25) examined the effects of the Halo Sport System of tDCS on the endurance of elite rowers in the same rowing ergometer model. They placed the electrodes on the CZ area (CZ-M1), and bilateral cathodal stimulators were set over C5 and C6 at 2.2 mA for 20 min (30 s for the sham group). The participants were required to perform 5 km of rowing with a constant load at a 20 stroke/rate, increasing their time and power by each 500 m/split until they had completed 5,000 m. They found differences in time (2,500 and 4,000 m) and power (at all distances measured except 3,000 and 3,500 m). According to the authors, these results can be explained by the rowers performing under the verbal encouragement of their performance coaches. This volitional action helped them achieve optimal performance results, in contrast to the present study, where the motivation came from the researchers.

Ma et al. (26) aimed to reach two objectives: first, to investigate the central mechanisms of tDCS in improving the male rower's performance; and second, to examine whether the improvements were related to the changes in the brain and topological activity characteristics of the functional brain network using resting-state functional MRI (rs-fMRI). They performed separate stimulations per group (1 or 2 mA) targeting LM1 (20 min, five times weekly for 2 weeks). They established that simultaneous tDCS-induced excitations over the M1 might improve the male rower's overall performance through the right precentral gyrus, left paracentral lobule, and left inferior frontal gyrus. Those areas initiate voluntary movement, while the inferior frontal gyrus is a crucial area of the cognitive–motor network, vital for planning motor actions (26). The authors should have stated where the cathode electrode was placed and shared the quadriceps and latissimus dorsi performance using the isokinetic dynamometer.

Other results demonstrated a reduced RPE after tDCS was followed by a cycle time to task failure in an incremental ramp test performed on the cycle ergometer. The results demonstrated that the anodal tDCS condition effectively increased exercise tolerance in the cycle ergometer at 100% peak power (18). Nonetheless, da Silva Machado et al. (21) enrolled seven cyclists and five rowers to test whether a new tDCS technique called high-definition tDCS at 2.4 mA would improve exercise performance to a greater extent than conventional tDCS (2.0 mA). The stimulation was conducted for 20 min before performing the time to exhaustion in cycling. The authors found no effect of the tDCS application on exercise performance or subjective responses to exhaustive exercise. They argued that these results contrast with those of the meta-analysis provided by Machado et al. (43), which showed a significant effect inflated by a single study [by Vitor-Costa et al. (38)]. The study by Vitor-Costa et al. (38) presented a more considerable weight (85.5%) over the other studies in Machado et al. (43), which can be explained by the lower variance given.

Nevertheless, most contemporary literature has ignored the importance of the brain in regulating exercise performance (18). Sport neuromodulation is a new area; much information is to be taken (53). Despite this, as mentioned, Amann et al. (14) doubt the actual effect of tDCS on corticospinal excitability and the improvement of sports performance and fatigability. Despite this, some researchers postulate that tDCS has been recently used before exercise to improve exercise performance in different types of exercise (18), but this information must be interpreted with caution (3, 14, 21, 54–56). Establishing a cause-and-effect relationship between brain responsiveness and exercise performance is difficult (14).

Lastly, the present study has some limitations. The number of participants should be increased. Unfortunately, the restrictions would involve the control of the competitive and training loads to understand the participants' individual external and internal loads to better understand their performance at this study test (in a competitive period undergoing regular exercise). In addition, the participants’ diet should be evaluated using an individualized and flexible nutrition assessment, for instance, to check the participants’ consumption of caffeine or other psychostimulant beverages. The blinding control is crucial to finding a possible placebo effect in the participants. Further, the participants’ sleep routines should also be assessed. It is essential to check the anthropometric variables once it is known that elite rowers are taller, heavier, and have better upper and lower limb lengths, breadths, and girths (3, 21, 56). In addition, despite the aforementioned information, we should be aware that “the perceived exertion, or the subjective experience of how hard a physical task feels, is quite different from perceived fatigue, which we argue is a feeling of diminishing capacity to cope with physical or mental stressors, either imagined or real…” (57). In this sense, we should be careful to interpret the real fatigue sensation using a subjective scale. In future studies, it is important to add different tools to understand the perceived level of fatigue. Further, we should try to check different tDCS montages once the efficacy of the T3-Fp2 montage seems questionable (53). Contrary as mentioned (53) Barwood et al. (54) did not assess the prefrontal cortex or perform any tests to confirm this misunderstanding. Perhaps the detailed montage, as presented in this study, would be attractive (40). This reference was not described in many studies that looked for the LIC. However, using electroencephalography and/or fMRI to confirm activation of the targeted area or another area with an exquisite machine, such as the Halo Sport System of tDCS or high-definition tDCS, or even a transcranial magnetic stimulation with or without a combination of tDCS, could be interesting. Some prefrontal tests to check the executive functions would also be necessary to confirm the possible influence of the cathodal electrode at the Fp2 as described by Ciccone et al. (53).

### Practical applications

This study may provide helpful preliminary insights related to this protocol design that can be used in future studies. The present results based on the current data suggest that the tDCS anodal LIC combined with a cathodal Fp2 montage would be effective in targeting the insular cortex. Thus, the use of anodal tDCS in the temporal lobe influences exercise capacity. However, plenty of unanswered questions are to be studied related to tDCS and exercise performance. Many tDCS montages and physical, physiological, and subjective variables can be explored. The inconsistency in the literature may be due to the methodologic setup. Indeed, these discrepancies highlight the need for more research on tDCS and exercise performance (53). Nevertheless, tDCS and sports performance are up-and-coming areas for the club and the coach's investment. This investment should be based on several scientific articles and their purpose regarding stimulation and performance.

## Data Availability

The raw data supporting the conclusions of this article will be made available by the authors, without undue reservation.
